# Metagenomic profiling of tobacco root endophytes reveals a disease-suppressive *Enterobacter* strain against *Fusarium solani*

**DOI:** 10.3389/fmicb.2026.1924993

**Published:** 2026-08-25

**Authors:** Tianmiao Li, Xiaoyu Zhou, Fei Xiong, Tianfu Zhao, Ning Jiang, Yongzhan Cai, Yanxia Hu, Canhua Lu, Yuanhu Xuan, Xiaotong Gai

**Affiliations:** 1 Yunnan Academy of Tobacco Agricultural Sciences, Kunming, China; 2 State Key Laboratory of Elemento-Organic Chemistry and Department of Chemical Biology, National Pesticide Engineering Research Center (Tianjin), Nankai University, Tianjin, China; 3 Dali Tobacco Company of Yunnan, Dali, China; 4 Qujing Tobacco Company of Yunnan, Qujing, China

**Keywords:** *Enterobacter*, *Fusarium solani*, metagenomics, root endophytes, sugar transporter, tobacco root rot

## Abstract

**Background:**

Tobacco root rot, caused by *Fusarium* species, is a persistent soil-borne disease that threatens tobacco production. To identify endophytic contributors to disease suppression, this study compared the root endophytic microbiomes of healthy and diseased tobacco plants using metagenomic sequencing and isolated functional bacteria from healthy roots.

**Results:**

Metagenomic analysis of 30 root samples (223 Gb) generated 2.9 million non-redundant genes and identified 1,953 core genera. Healthy plants contained distinct endophytic microbial communities enriched in bacterial taxa and pathways associated with secondary metabolite biosynthesis, siderophore production, chemotaxis, biofilm formation, and carbohydrate metabolism. This microbiome-guided approach identified TM-1, an endophytic *Enterobacter* strain that significantly inhibited *Fusarium solani* by 62.29% in a dual-culture assay. Transcriptome profiling revealed that TM-1 treatment broadly altered *F. solani* gene expression, with prominent effects on ribosome function, amino acid biosynthesis, carbon metabolism, and glycolysis. TM-1 disrupted sugar transporter-related gene expression, and deletion of five representative genes significantly restricted fungal mycelial growth, with the strongest inhibition (66.47%) observed for the hexose transporter homolog MRS44_010803.

**Conclusion:**

These results indicate that healthy tobacco roots harbor disease-suppressive endophytic microorganisms and suggest that TM-1 may suppress *F. solani* by interfering with sugar transport and carbon acquisition. These findings provide a potential biocontrol resource for the sustainable management of tobacco root rot.

## Introduction

Tobacco root rot, a globally distributed soil-borne disease caused by *Fusarium* species, is prevalent in major tobacco-producing regions of China, including Yunnan, Guizhou, Hunan, and Sichuan (Berruezo et al., 2018; Yang et al., 2020). The pathogens typically enter tobacco plants through the roots and colonize plant tissues, causing wilting, plant death and severe reductions in tobacco yield and quality (Berruezo et al., 2018; Gao et al., 2021). The pathogens of tobacco root rot can persist in soil and plant residues for long periods through the formation of chlamydospores and the maintenance of mycelial structures. This long-term survival capability and cryptic infection strategy increase disease management difficulty and limit the effectiveness of conventional control practices (Yang et al., 2025). Although resistant cultivars can reduce disease incidence to some extent, stable resistance is difficult to maintain in complex pathogen populations. Therefore, integrated, effective, and sustainable disease management strategies are needed for tobacco root rot.

During long-term co-evolution, plants and diverse microorganisms have established stable interactions (Bulgarelli et al., 2013). Based on their colonization niche, root-associated microorganisms are generally classified as rhizosphere microorganisms or root endophytes (Lundberg et al., 2012). These microorganisms can enhance plant disease resistance and promote plant growth through various mechanisms, including niche competition, production of antimicrobial metabolites, and induction of plant systemic resistance (Berendsen et al., 2012; Pieterse et al., 2014; Compant et al., 2019; Al-Ani et al., 2020). Increasing evidence indicates that under pathogen infection, plants can alter root exudate composition to selectively recruit beneficial microorganisms for defense, a phenomenon known as the “cry for help” strategy (Yuan et al., 2018; Rolfe et al., 2019). Thus, manipulating plant-associated microbial communities to establish disease-suppressive microbial environments represents an important approach for sustainable disease management (Compant et al., 2025; Hajji-Hedfi et al., 2025).

Extensive studies have revealed the important role of rhizosphere microorganisms in the control of soil-borne diseases. Beneficial microorganisms such as *Bacillus* species can inhibit pathogen growth through antimicrobial metabolites or enhance plant disease resistance by inducing host immunity (Ongena and Jacques, 2008; Yu et al., 2011; Fazle Rabbee and Baek, 2020). However, compared with rhizosphere microorganisms, the role of root endophytes in plant defense against soil-borne diseases remains less explored. Rhizosphere microorganisms mainly colonize the soil–root interface, whereas root endophytes can enter and persist within internal plant tissues. Due to their close association with soil-borne pathogen infection processes, root endophytes may restrict pathogen colonization and enhance plant resistance (Hardoim et al., 2008; Reinhold-Hurek and Hurek, 2011; Aydi-Ben-Abdallah et al., 2020; Hajji-Hedfi et al., 2026). Therefore, understanding the contribution of root endophytes to disease suppression represents an important step toward developing green control approaches against *Fusarium* diseases. In this study, we aimed to elucidate the association between root endophytic microbiomes and tobacco root rot suppression and identify beneficial microorganisms associated with plant health.

## Materials and methods

### Sample collection and processing

A five-point sampling method was used. In each sampling plot, three healthy tobacco plants and three diseased tobacco plants were randomly selected. The study included two groups: a healthy plant group and a diseased plant group. Each group comprised five biological replicates, with each replicate consisting of three tobacco plants. In total, 30 root tissue samples were obtained, including 15 samples from healthy plants and 15 samples from diseased plants. Root tissues were surface sterilized to remove environmental microorganisms and root surface-associated microbes. The surface-sterilized root tissues were cut into small pieces with a sterile scalpel in a laminar flow hood, snap-frozen in liquid nitrogen, and stored at −80 °C until further use.

### DNA extraction of root endophytic microorganisms

For the 30 root tissue samples, the surface-sterilized tissues were thoroughly ground to a fine powder in liquid nitrogen before DNA extraction using the TIANamp Bacteria DNA Kit (TIANGEN, China) according to the manufacturer’s instructions. DNA concentration and purity were determined using a NanoDrop 2000 spectrophotometer, with OD_260/280_ values of 1.8–2.0 and OD_260/230_ values greater than 1.8. DNA quality was assessed using 1% agarose gel electrophoresis.

### Metagenomic sequencing of root endophytic microorganisms

To characterize the composition and functional features of endophytic microbiomes in healthy and diseased tobacco roots, metagenomic sequencing was performed using the extracted DNA of root endophytic microorganisms. DNA samples that passed quality control were used for paired-end library construction. Libraries were constructed using the TruSeq Nano DNA Library Prep Kit (Illumina), with an average insert size of approximately 350 bp. Fragmented DNA underwent end repair, 3′-end adenylation, adapter ligation, and 8 cycles of PCR enrichment. Size selection was performed using Agencourt AMPure XP beads (Beckman Coulter, USA). Library concentration was quantified by qPCR using the KAPA Library Quantification Kit (Kapa Biosystems, USA) on a Roche LC480 instrument. The indexed libraries were pooled and subjected to paired-end sequencing (2 × 150 bp) on an Illumina sequencing platform at Shanghai OE Biotech Co., Ltd.

### Metagenomic sequencing data analysis

Raw metagenomic sequencing data were generated in FASTQ format. Adapter sequences and low-quality bases were removed using Trimmomatic (v0.36), and reads shorter than 50 bp or with an average base quality below 20 were discarded. The quality-filtered reads were mapped to the tobacco reference genome using Bowtie2, and the host-derived sequences were removed to obtain clean microbial reads. The filtered microbial reads were assembled using MEGAHIT (v1.1.2), and contigs longer than 500 bp were retained for downstream analysis. Open reading frames were predicted using Prodigal (v2.6.3) in metagenomic mode, and the predicted genes were translated into amino acid sequences. All predicted genes were clustered using CD-HIT (v4.6.7) to construct a non-redundant gene catalog. Clustering was performed at thresholds of 95% sequence identity and 90% alignment coverage, with the longest sequence in each cluster selected as the representative sequence. Taxonomic annotation was performed using the NR database. The representative amino acid sequences from the non-redundant gene catalog were aligned against the NR database using DIAMOND (v2.0.13). Clean reads from each sample were mapped to the non-redundant gene catalog using Bowtie2 (v2.2.9) at a similarity threshold of 95%, followed by the calculation of gene abundance for each sample. For functional annotation, representative amino acid sequences from the non-redundant gene catalog were aligned against the NR, KEGG, COG, and eggNOG databases using DIAMOND. The E-value threshold for all database searches was set to 1e^−5^. The resulting annotations were used to analyze the taxonomic composition, functional gene profiles, and metabolic pathways of the microbial communities.

### Isolation and purification of TM-1

Endophytic bacteria were isolated from tobacco root tissue samples using the dilution plating method. Single colonies with distinct morphologies and vigorous growth were selected and repeatedly streaked on LB medium at least three times to obtain pure cultures. The purity of the isolates was confirmed by repeated streaking until uniform colony morphology was observed. The purified bacterial isolates were cultured on LB medium at 30 °C for 12 h before preservation. All purified isolates were preserved as glycerol stocks at a final glycerol concentration of 15–20% and stored at −80 °C.

### Morphology identification

The morphological characteristics of the purified bacterial isolates were examined by observing colony morphology on LB agar plates. Activated bacterial strains were inoculated into 100 mL of liquid LB medium and cultured at 28 °C with shaking at 200 rpm for 24 h. The bacterial cultures were serially diluted 10-fold seven times under aseptic conditions, and 100 μL of the diluted suspensions was spread onto LB agar plates. During bacterial growth, colony characteristics, including color, shape, size, margin, elevation, and surface texture, were recorded for preliminary morphological identification of the antagonistic strains.

### Molecular method and construction of phylogenetic tree

Genomic DNA was extracted from each isolate using the TIANamp Bacteria DNA Kit (DP302, TIANGEN, China) according to the manufacturer’s instructions. The 16S rRNA gene was amplified using universal bacterial primers 27F: AGAGTTTGATCMTGGCTCAG and 1492R: TACGGYTACCTTGTTACGACTT. PCR amplification was performed in a 25 μL reaction mixture containing 1 μL of forward primer (10 μM), 1 μL of reverse primer (10 μM), 12.5 μL of 2 × Phanta Flash Master Mix (P520, Vazyme, China), 1 μL of template DNA, and sterile deionized water to a final volume of 25 μL. The PCR amplification was conducted under the following conditions: initial denaturation at 98 °C for 30 s; followed by 35 cycles of denaturation at 98 °C for 10 s, annealing at 58 °C for 10 s, and extension at 72 °C for 10 s; with a final extension at 72 °C for 1 min. The PCR products were verified by agarose gel electrophoresis and submitted for sequencing. The obtained sequences were compared with the NCBI database using BLAST. Furthermore, the sequences of closely related type strains were selected for phylogenetic analysis, and a phylogenetic tree was constructed using the neighbor-joining method in MEGA X with 1,000 bootstrap replicates.

### Dual-culture antagonism assay

As the causal agent of tobacco root rot, *F. solani* was used as the target pathogen in the dual-culture antagonism assay. The *F. solani* strain used in this study was obtained from the Department of Chemical Biology, College of Chemistry, Nankai University, and maintained in our laboratory before the experiments. The pathogen was first grown on PDA plates at 28 °C for 3–5 d. Subsequently, a 5-mm mycelial plug was excised from the actively growing colony margin using a sterile cork borer and placed upside down at the center of a 9-cm PDA plate. The candidate antagonistic bacterial strains were grown in LB broth to logarithmic phase, and cell suspensions were adjusted to 1 × 10^8^ CFU/mL. For bacterial inoculation, four points were arranged symmetrically 2.5 cm from the pathogen plug, and 5 μL of bacterial suspension was applied to each point. The control plates were inoculated with only the pathogen plug. All plates were incubated at 28 °C for 5–7 d. After 3 d, the pathogen colony diameter was measured for each treatment, and the inhibition rate was calculated as follows:


$$\;\text{Inhibition rate}\;(\%)=\frac{\left(\begin{array}{l} \text{colony diameter of control}\\ -\text{colony diameter of treatment} \end{array}\right)}{\text{colony diameter of the control}}\times 100\%$$

Each treatment included three biological replicates, and the experiment was repeated three times.

### Transcriptome profiling of *Fusarium solani* exposed to TM-1

To examine the mechanisms underlying the TM-1-mediated inhibition of *F. solani* growth, RNA-seq analysis was conducted on *F. solani* exposed to TM-1 treatment. The antagonistic strain TM-1 was inoculated into LB broth and cultured at 30 °C and 180 rpm to logarithmic phase (OD_600_ = 0.8). The culture supernatant was collected and passed through a 0.22-μm sterile membrane to obtain the TM-1 cell-free filtrate. The filtrate was incorporated into the PDA medium at final proportions of 10 and 20% to prepare TM-1-treated media, while PDA medium without filtrate was used as the control. A 5-mm mycelial plug of *F. solani* was placed at the center of each plate and incubated at 28 °C. Mycelia from the treatment and control groups were collected after 18 and 36 h of incubation, immediately snap-frozen in liquid nitrogen, and stored at −80 °C. Total RNA was extracted using TRIzol reagent for transcriptome library construction and sequencing.

### Transcriptome library construction, sequencing, and data preprocessing

For transcriptome library construction, 1 μg of total RNA was used from each sample. Moreover, mRNA was enriched with oligo(dT) magnetic beads, fragmented, and reverse-transcribed into double-stranded cDNA using random primers. cDNA libraries were generated through end repair, dA-tailing, adaptor ligation, fragment size selection, and PCR amplification, and qualified libraries were sequenced on the Illumina NovaSeq 6000 platform to produce paired-end 150-bp reads. Raw FASTQ reads were filtered using a Phred quality threshold of 20, a minimum retained read length of 75 bp, and a maximum allowance of five ambiguous bases. Clean reads were mapped to the *F. solani* reference genome from NCBI using HISAT2 (v2.2.1). Only samples with mapping rates greater than 70% were retained for downstream analysis, and the alignment outputs were saved as sorted BAM files.

### Transcriptome data analysis and functional annotation

Gene-level read counts were obtained from BAM files using HTSeq (v0.6.1) and assembled into a gene-by-sample count matrix. Differential expression analysis was conducted using DESeq2 (v1.34.0), which normalized the read counts using the median-of-ratios method and tested differential expression using the Wald test. The Benjamini–Hochberg procedure was applied for multiple testing corrections. Genes were defined as differentially expressed when they met both thresholds of absolute log_2_ fold change (|log_2_FC|) ≥ 1 and false discovery rate (FDR) < 0.05. To evaluate sample reproducibility, principal component analysis (PCA) was performed on FPKM values using the *prcomp* function in R. Furthermore, differentially expressed genes (DEGs) were analyzed for functional enrichment using Gene Ontology (GO) and Kyoto Encyclopedia of Genes and Genomes (KEGG) annotations. GO enrichment analysis was performed using GOSeq (v1.34.1), with the gene length bias corrected using the Wallenius approximation method. GO terms with FDR < 0.05 were considered significant and visualized using topGO. KEGG pathway enrichment was assessed using a hypergeometric test with an FDR threshold of 0.05, and the enrichment results were displayed as bar plots. Gene set enrichment analysis (GSEA) was performed with gseapy (v1.1.9) using edgeR-normalized counts per million (CPM) as input, retaining genes with CPM > 1 in at least 50% of the samples. Gene sets from the MSigDB Hallmark collection, GO, and KEGG databases were included, and genes were ranked by signal-to-noise ratio. After 1,000 permutations, gene sets with FDR < 0.25 were considered significantly enriched. Leading-edge analysis was used to identify the core genes that contributed most strongly to enrichment signals.

### Construction of knockout mutants in *Fusarium solani*

The deletion mutant of the target gene in *F. solani* was generated using a split-marker strategy as previously described (Scala et al., 2017). Briefly, approximately 1 kb upstream and downstream flanking regions of the target gene were amplified from the genomic DNA of the wild-type strain using specific primer pairs (Supplementary Table S8). The two flanking fragments were fused with two overlapping fragments of the hygromycin resistance cassette by fusion PCR to generate the split-marker deletion constructs. Protoplasts were prepared from actively growing mycelia treated with cell wall-degrading enzymes, and the deletion constructs were introduced into *F. solani* protoplasts by PEG-mediated transformation (Goswami, 2012). Transformants were selected on PDA plates containing hygromycin B (30 μg mL^−1^). Hygromycin-resistant colonies were screened by PCR using primers flanking the target gene region and further confirmed by DNA sequencing.

### Statistical analysis

Statistical comparisons were performed according to the number of groups and data distribution. For normally distributed data, two-group comparisons were performed using Student’s *t*-test, whereas comparisons among multiple groups under a single variable were conducted using one-way analysis of variance (ANOVA) followed by Tukey’s *post hoc* test. For data that did not satisfy the normality assumption, the Wilcoxon rank-sum test was used for two-group comparisons, and the Kruskal–Wallis test followed by Dunn’s post hoc test was used for multiple-group comparisons. All tests were two-tailed, with *p* < 0.05 considered statistically significant. Differential taxa and functional features were identified using linear discriminant analysis effect size (LEfSe), with thresholds of LDA score > 2 and *p* < 0.05. The microbial community structure was compared using analysis of similarities (ANOSIM) and permutational multivariate analysis of variance (PERMANOVA). Routine statistical analyses and data visualization were performed using GraphPad Prism 8.

## Results

### Metagenomic sequencing generates a comprehensive gene catalog of tobacco root endophytic microbiomes

Metagenomic sequencing was conducted on 30 tobacco root samples to characterize the differences in root endophytic microbiomes between healthy and diseased plants. Sequencing generated 223 Gb of raw data, of which approximately 217 Gb were retained as high-quality reads after filtering. The clean data per sample ranged from 6.23 to 9.09 Gb, providing sufficient sequencing depth for downstream metagenomic analyses. After assembly, the total length of assembled sequences varied from 6,212,431 to 204,429,758 bp among samples, with N50 values of 662–1,050 bp and N90 values of 521–556 bp. The coding sequences predicted from the assembled contigs were clustered at 95% sequence identity, producing a non-redundant gene catalog containing 2,896,902 genes and an average open reading frame length of 608.96 bp. Core-pan analysis was used to determine whether the sampling effort adequately represented the genetic repertoire of the tobacco root endophytic microbiome. With increasing sample numbers, both the pan- and core-genome curves gradually approached a plateau, indicating that the current sampling scale was sufficient to capture the core genetic repertoire and support downstream functional analyses. Additionally, the non-redundant gene catalog was annotated against the KEGG, eggNOG, NR, and SWISS-PROT databases, yielding annotation rates of 28.62–92.59%. These results established a comprehensive metagenomic dataset for tobacco root endophytic microorganisms and provided a foundation for subsequent taxonomic and functional analyses of root endophytic microbiomes between healthy and diseased tobacco plants. The assembled sequence characteristics of individual samples are summarized in Supplementary Table S1.

### Distinct community composition of root endophytic microorganisms between healthy and diseased tobacco plants

**Figure 1:**
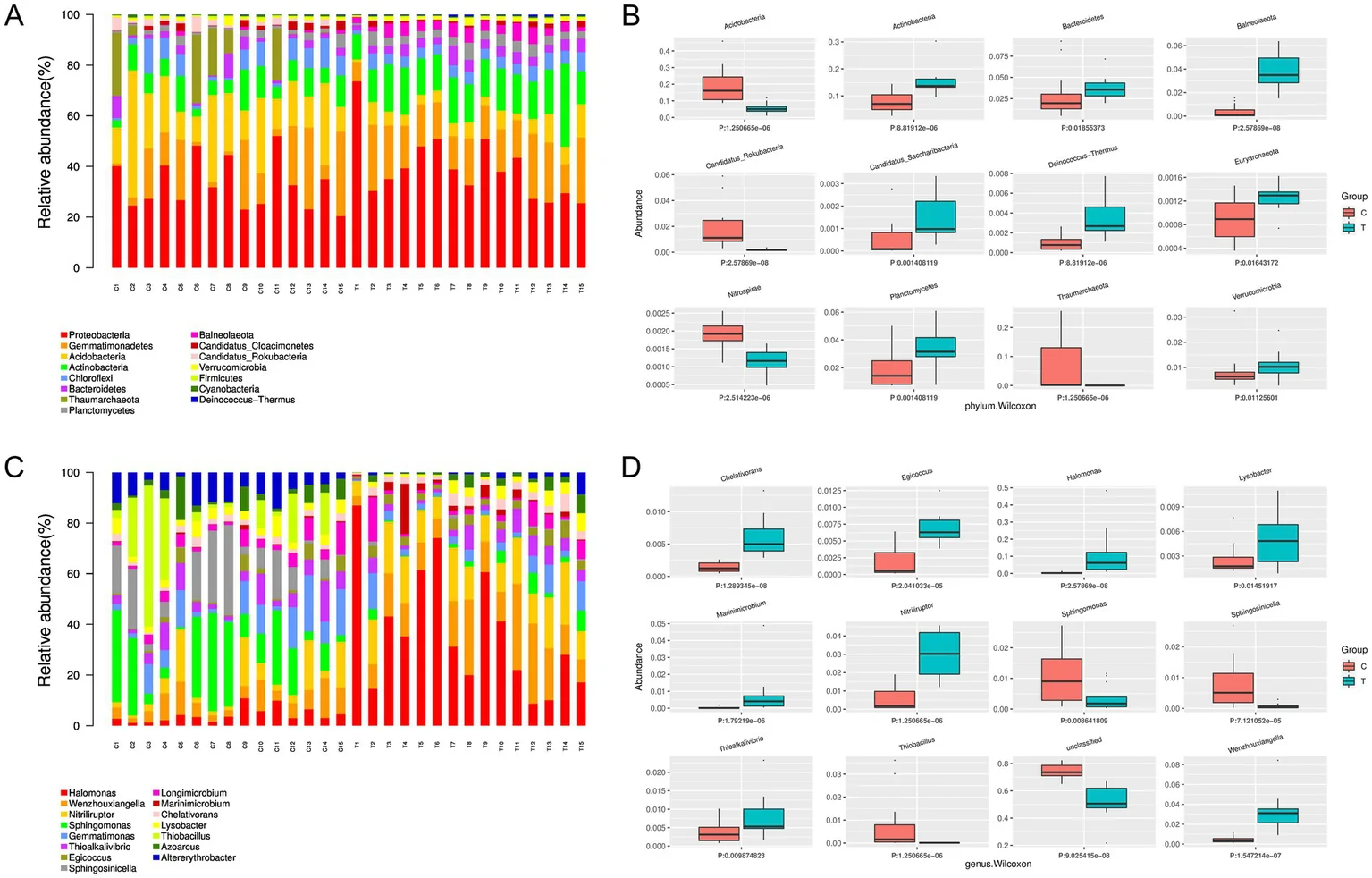
Distinct taxonomic profiles of root endophytic bacterial communities in healthy and diseased tobacco plants. **(A)** Relative abundance of root endophytic bacterial communities at the phylum level. **(B)** Top 12 bacterial phyla showing significant differences in relative abundance between healthy (T) and diseased (C) tobacco plants, as determined by the Wilcoxon rank-sum test. **(C)** Relative abundance of root endophytic bacterial communities at the genus level. **(D)** Top 12 bacterial genera showing significant differences in relative abundance between healthy (T) and diseased (C) tobacco plants, as determined by the Wilcoxon rank-sum test. Statistical significance was set at *p* < 0.05.

In addition to Krona visualization, taxonomic annotation based on the NR database was used to characterize the composition of tobacco root endophytic microorganisms across taxonomic levels. Bacteria accounted for most annotated sequences in all samples, with an average proportion of 92.01%. Archaea, Eukaryota, viruses, and other unclassified taxa accounted for smaller proportions (Supplementary Table S2). Therefore, subsequent analyses focused mainly on root endophytic bacterial communities. A comparison of endophytic bacterial communities between healthy and diseased tobacco roots identified 192 bacterial phyla across the 30 samples. The dominant phyla were Proteobacteria, Gemmatimonadetes, Acidobacteria, Actinobacteria, and Chloroflexi (Figure 1A). Proteobacteria was the most abundant phylum, accounting for an average relative abundance of 33.19% across all samples, followed by Gemmatimonadetes at 14.15%. Significant differences in phylum-level composition were observed between the two plant health states. Actinobacteria and Bacteroidetes were significantly more abundant in healthy roots than in diseased roots, increasing by 96.76 and 30.69%, respectively (Figure 1B). In contrast, Acidobacteria was 71.39% lower in healthy roots than in diseased roots (Figure 1B). These results indicate that tobacco root rot is associated with pronounced shifts in the root endophytic bacterial community composition. Community-level differences were further supported by beta-diversity analysis. PCoA separated healthy and diseased samples along the first principal coordinate, although several samples partially overlapped (Supplementary Figure S1A). ANOSIM analysis confirmed a significant difference in the genus-level community structure between the two groups (*R* = 0.604, *p* = 0.001) (Supplementary Figure S1B). At the genus level, 3,339 bacterial genera were detected across all samples. *Halomonas*, *Wenzhouxiangella*, and *Nitriliruptor* were the three most abundant genera, with average relative abundances of 5.47, 1.80, and 1.80%, respectively (Figure 1C). Among the genera enriched in healthy plants, *Halomonas* presented the largest increase, rising from 0.31% in diseased plants to 10.64% in healthy plants, followed by *Wenzhouxiangella* and *Nitriliruptor*. Conversely, *Sphingomonas* and *Thiobacillus* were significantly more abundant in diseased plants than in healthy plants (Figure 1D). LEfSe analysis further identified taxa that differentiated the two groups. *Chelativorans*, *Egicoccus*, *Halomonas*, *Marinimicrobium*, *Nitriliruptor*, and *Wenzhouxiangella* were significantly enriched in healthy roots (LDA > 2, *p* < 0.05), whereas *Thiobacillus*, *Lysobacter*, *Thioalkalivibrio*, *Sphingomonas*, and *Sphingosinicella* were significantly enriched in diseased roots relative to healthy roots (Supplementary Figure S2). Overall, these results indicate that healthy and diseased tobacco plants maintain distinct root endophytic bacterial communities. The differences were mainly reflected in the shifts in the dominant bacterial phyla and genera, with several bacterial taxa showing differential enrichment between healthy and diseased roots.

### Functional differences of root endophytic microbiomes between healthy and diseased tobacco plants

**Figure 2:**
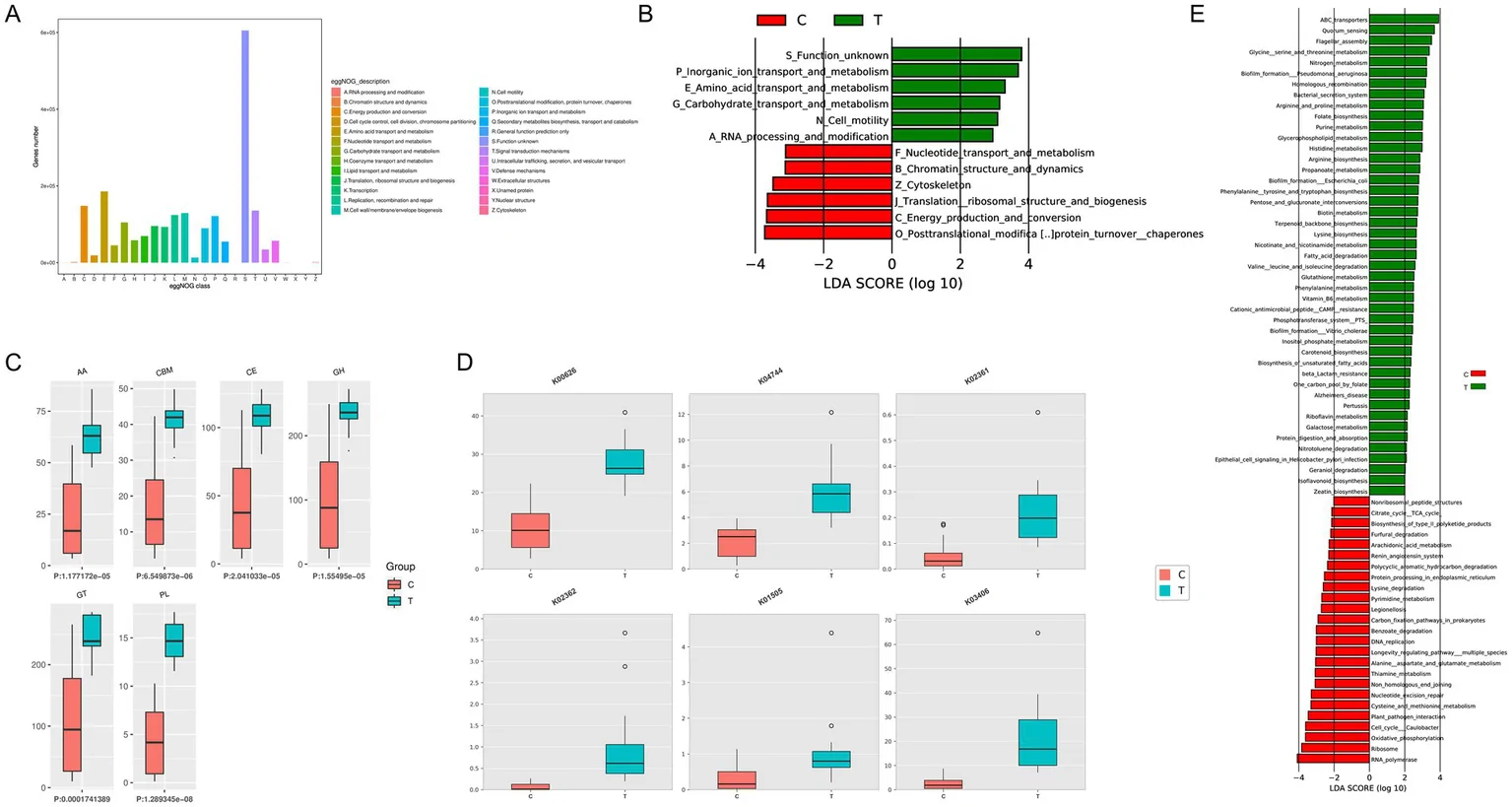
Functional differences in root endophytic bacterial communities between healthy and diseased tobacco plants. **(A)** Distribution of functionally annotated genes based on the eggNOG database. **(B)** Differential eggNOG functional categories identified by LEfSe analysis (LDA score > 2). **(C)** Differentially abundant CAZy functional categories between healthy and diseased plants. **(D)** Differentially abundant KEGG functional categories between healthy and diseased plants. **(E)** Differential KEGG level 3 pathways identified by LEfSe analysis (LDA score > 2). Statistical significance was set at *p* < 0.05.

The functional annotation of the metagenomic non-redundant gene catalog revealed functional differences between root endophytic microbiomes from healthy and diseased tobacco plants. Annotation against the eggNOG database assigned 2,184,107 non-redundant genes to COG functional categories, corresponding to an annotation rate of 74.21% (Supplementary Table S3). The COG functional profiles differed significantly between healthy and diseased root endophytic microbiomes (Figure 2A). A comparison between the two plant health states revealed distinct COG functional profiles. In healthy plants, genes assigned to inorganic ion transport and metabolism (COG_P), amino acid transport and metabolism (COG_E), carbohydrate transport and metabolism (COG_G), cell motility (COG_N), and RNA processing and modification (COG_A) were significantly enriched compared with diseased plants (LDA score > 2) (Figure 2B). In diseased plants, the enriched categories shifted toward nucleotide transport and metabolism (COG_F), chromatin structure and dynamics (COG_B), cytoskeleton (COG_Z), translation, ribosomal structure and biogenesis (COG_J), energy production and conversion (COG_C), and post-translational modification, protein turnover, and chaperones (COG_O) (LDA score > 2) (Figure 2B). Moreover, carbohydrate-active enzyme annotations separated the two groups. CAZy analysis assigned 58,134 non-redundant genes to carbohydrate-active enzyme families. Glycosyl transferases (GTs), glycoside hydrolases (GHs), and carbohydrate esterases (CEs) represented the major CAZy categories (Supplementary Figure S3). Differential abundance analysis demonstrated that all major CAZy categories, including GHs, GTs, polysaccharide lyases (PLs), CEs, auxiliary activities (AAs), and carbohydrate-binding modules (CBMs), were significantly more abundant in the root endophytic microbiome of healthy plants than in that of diseased plants (Figure 2C). These results indicate that healthy and diseased plants exhibited distinct carbohydrate-active enzyme profiles within their root endophytic microbiomes.

KEGG annotation was used to identify functional modules associated with differences between healthy and diseased root endophytic microbiomes. In total, 28.62% of the non-redundant coding genes were assigned to specific functional annotations, generating 7,922 KOs (KEGG Orthology). The most abundant KO was K02004, annotated as putative ABC transport system permease protein (Supplementary Figure S4), indicating that ABC transport-related functions represented abundant functional components in the tobacco root endophytic microbiome. In healthy plants, the significantly enriched KOs were concentrated in secondary metabolism and natural product biosynthesis, including functions related to secondary metabolism, natural product biosynthesis, and microbial interaction. KOs associated with 1-aminocyclopropane-1-carboxylate deaminase (K01505, acdS) and methyl-accepting chemotaxis protein (K03406, mcp) were significantly enriched in healthy plants (Figure 2D). These results indicate differences in secondary metabolism, stress-related functions, and bacterial chemotaxis between healthy and diseased root endophytic microbiomes.

Furthermore, the KO abundance profiles were summarized at the KEGG Level 3 pathway level, yielding 236 third-level functional pathways (Supplementary Table S4). LEfSe analysis (LDA > 2.0) identified the pathways that contributed most strongly to the functional differentiation between the two groups. In healthy plants, enriched pathways included flagellar assembly, zeatin biosynthesis, biofilm formation, and isoflavonoid biosynthesis (Figure 2E). In contrast, diseased plants showed enrichment of plant–pathogen interaction and legionellosis-related pathways (Figure 2E). Overall, these results demonstrate that healthy and diseased tobacco plants harbored distinct functional profiles of root endophytic microbiomes, with differences mainly observed in metabolism-related, transport-related, and microbial interaction-related pathways.

### Isolation and characterization of an antifungal *Enterobacter* strain TM-1 from healthy tobacco roots

**Figure 3:**
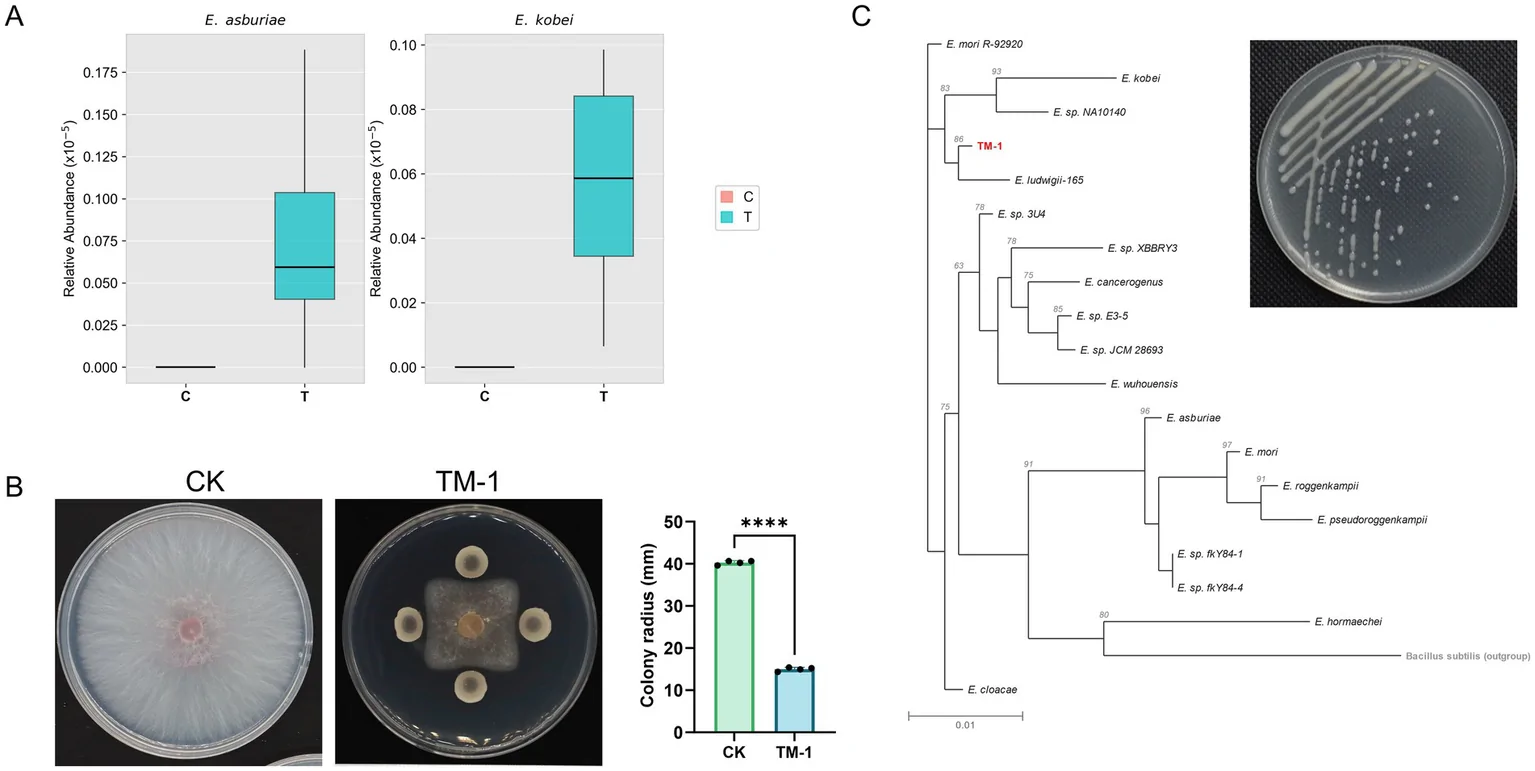
Identification and antagonistic activity of *Enterobacter* strain TM-1 against *F. solani*. **(A)** Relative abundances of *Enterobacter asburiae* and *Enterobacter kobei* in healthy and diseased tobacco roots. **(B)** Inhibitory effect of strain TM-1 on the mycelial growth of *F. solani* in dual-culture assay. The bars represent the colony radius of *F. solani*. Asterisks indicate significant differences relative to the control (^***^p < 0.0001). **(C)** Phylogenetic placement of strain TM-1 based on 16S rRNA gene sequence analysis. A phylogenetic tree was constructed using the neighbor-joining method with closely related *Enterobacter* species.

Core microbiome analysis was used to identify endophytic bacterial taxa differentially represented in healthy tobacco roots. Taxa detected in more than 80% of the samples were defined as core members, yielding 1,953 core genera (Supplementary Table S5). Within this core community, *Enterobacter* was detected in all samples at a detection rate of 100%, indicating that *Enterobacter* represented a core genus of the tobacco root endophytic microbiome. Species-level comparisons revealed differential enrichment within this genus. Notably, *E. asburiae* (*p* = 1.9 × 10^−5^) and *E. kobei* (*p* = 3.0 × 10^−6^) were significantly enriched in healthy root samples (Figure 3A), indicating enrichment of specific *Enterobacter* taxa in healthy roots. Based on these observations, culturable bacteria were isolated from healthy tobacco root tissues and screened for antifungal activity. Isolates were screened for antagonistic activity against *F. solani* using a dual-culture assay. Preliminary and secondary screenings yielded 20 strains with inhibitory activity against *F. solani*. Seven of these strains, TM-1, TM-7, TM-11, TM-13, TM-18, TM-19, and TM-20, exhibited strong antifungal activities, each with an inhibition rate greater than 50% (Supplementary Figure S5). TM-1 exhibited the strongest inhibitory effect, with an inhibition rate of 62.29% (Figure 3B). Therefore, TM-1 was selected for further characterization. Morphological observation on LB agar plates showed that TM-1 formed creamy-white, circular colonies with entire margins. The colonies were slightly raised, smooth, and moist, with a glossy surface. Subsequently, molecular identification was performed based on the full-length 16S rRNA gene sequence. The full-length 16S rDNA sequence was 1,422 bp, and BLAST comparison against the NCBI database, along with neighbor-joining phylogenetic analysis, placed TM-1 closest to *Enterobacter ludwigii*, with 99.86% sequence similarity (Figure 3C).

### TM-1 reprograms the transcriptome of *Fusarium solani*

**Figure 4:**
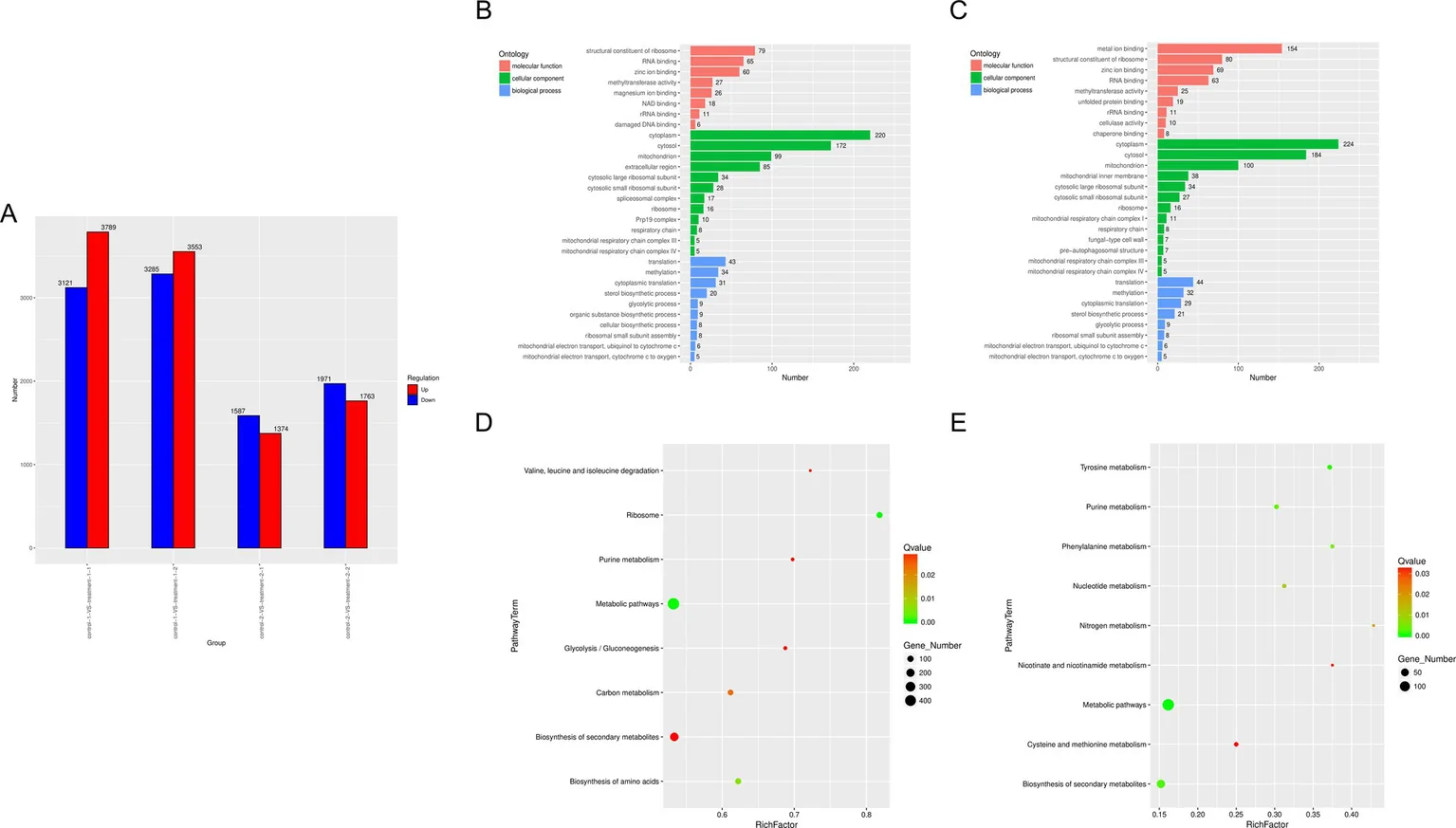
Transcriptomic responses of *F. solani* to TM-1 treatment. **(A)** Number of TM-1-upregulated and -downregulated differentially expressed genes in *F. solani* after TM-1 treatment. **(B)** GO enrichment analysis of differentially expressed genes after treatment with 10% TM-1 filtrate for 18 h. **(C)** GO enrichment analysis of differentially expressed genes after treatment with 20% TM-1 filtrate for 18 h. **(D)** KEGG pathway enrichment analysis of differentially expressed genes after treatment with 10 and 20% TM-1 filtrate for 18 h. **(E)** KEGG pathway enrichment analysis of differentially expressed genes after treatment with 10 and 20% TM-1 filtrate for 36 h.

RNA-seq was used to identify the transcriptional responses of *F. solani* associated with TM-1-mediated growth inhibition. *F. solani* mycelia were exposed to 10% or 20% TM-1 cell-free filtrate and collected after 18 and 36 h for transcriptome sequencing. Quality control generated 801,081,592 high-quality reads, with an average data yield of approximately 6.94 Gb per sample, an average Q30 base percentage of 93.22%, and GC content of 55.47–56.40%. Differential expression analysis was performed using FDR < 0.05 and |log_2_FC| ≥ 1 as thresholds. TM-1 exposure produced a strong early transcriptional response in *F. solani*. At 18 h, 6,910 and 6,838 DEGs were identified in the 10 and 20% TM-1 treatment groups, respectively (Figure 4A). Among these DEGs, 3,789 genes were upregulated and 3,121 genes were downregulated in the 10% treatment group, whereas 3,553 genes were upregulated and 3,285 genes were downregulated in the 20% treatment group (Figure 4A; Supplementary Figures S6A,B). By 36 h, the number of DEGs decreased to 2,961 in the 10% treatment group and 3,734 in the 20% treatment group (Figure 4A; Supplementary Figures S6C,D). These results indicate that TM-1 treatment induced stronger transcriptional changes at 18 h than at 36 h. GO enrichment analysis was used to define the functional features of these DEGs. The enriched terms covered biological process (BP), cellular component (CC), and molecular function (MF), comprising 117 subcategories in total. Upregulated genes were enriched in 105 subcategories, whereas downregulated genes were enriched in 87 subcategories (Supplementary Table S6). At 18 h, the 10 and 20% TM-1 treatments produced similar GO enrichment profiles. The main BP terms were translation, cytoplasmic translation, and glycolytic process and other biosynthetic processes. The main CC terms were cytosolic ribosomal subunit, ribosome, and cytoplasm, whereas the main MF terms were structural constituent of ribosome, methyltransferase activity, rRNA binding, RNA binding, and damaged DNA binding (Figures 4B,C). At 36 h, the GO enrichment signals were significantly weaker. After FDR correction, no significantly enriched GO terms were detected in either the 10% or 20% TM-1 treatment groups. These results suggest that the functional responses at 36 h differed from those observed at the early treatment stage.

KEGG enrichment supported the transcriptional responses induced by TM-1 treatment. At 18 h, DEGs were most strongly enriched in the ribosome pathway, together with the biosynthesis of amino acids, carbon metabolism, and glycolysis/gluconeogenesis (Figure 4D). At 36 h, the enriched pathways were mainly related to metabolic processes, secondary metabolite biosynthesis, and amino acid degradation (Figure 4E), suggesting distinct functional responses between early and late TM-1 treatments.

### Sugar transporter-related genes contribute to the growth inhibition of *Fusarium solani* by TM-1

**Figure 5:**
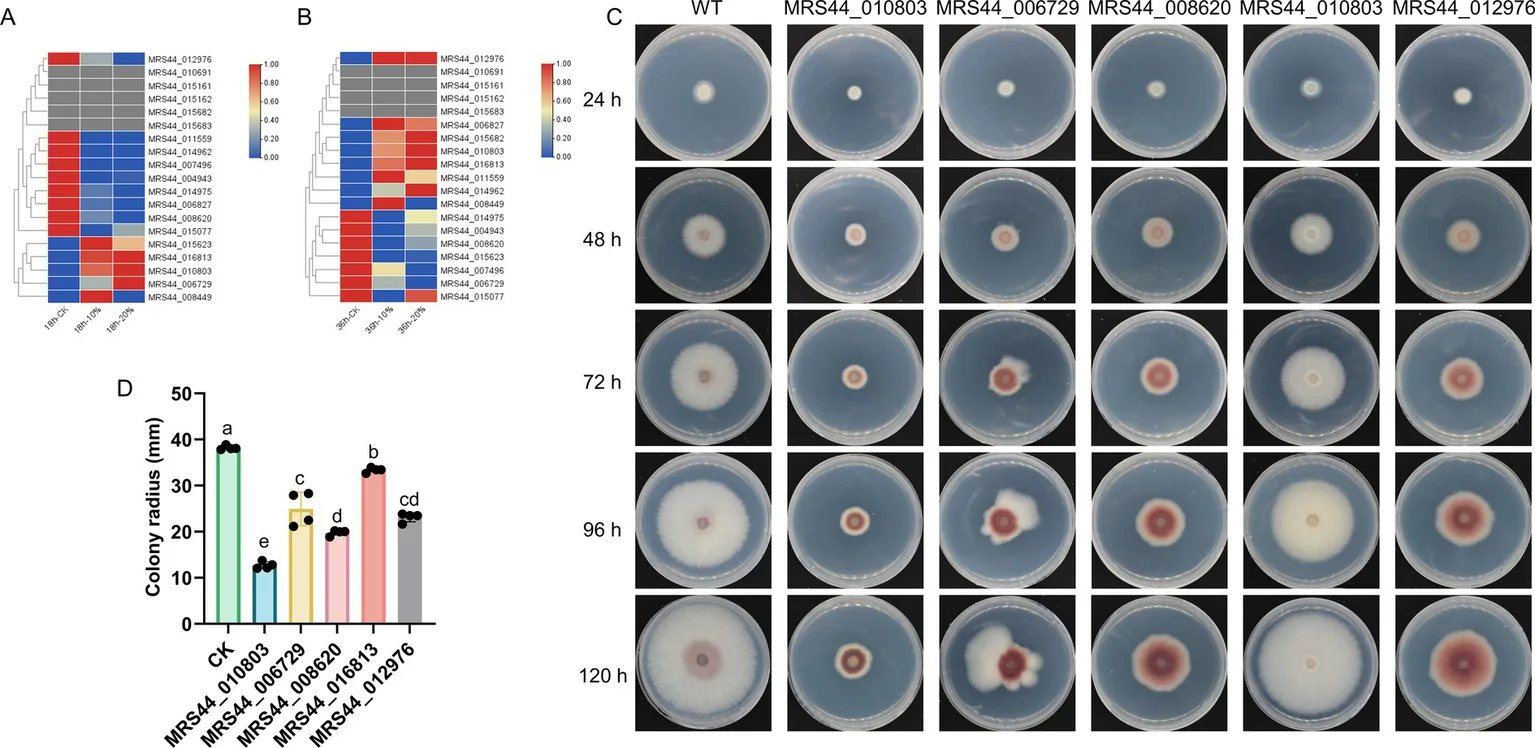
TM-1 treatment alters the expression of sugar transporter-related genes in *F. solani*, and deletion of these genes restricts fungal growth. **(A)** Heatmap showing the expression profiles of 19 sugar transporter-related genes after TM-1 treatment for 18 h. **(B)** Heatmap showing the expression profiles of 19 sugar transporter-related genes after TM-1 treatment for 36 h. **(C)** Colony morphology of sugar transporter gene deletion mutants and wild-type strain. **(D)** Quantification of mycelial growth of sugar transporter gene deletion mutants. Bars represent colony radius, and data are presented as the mean ± SD. Different letters indicate significant differences between treatments.

Transcriptome analysis identified 19 sugar transport-related genes across three sugar transport-related KO entries, K08139, K08141, and K08150. Functional annotation divided these genes into five classes, including one hexose transporter family (HXT) gene, 13 *α*-glucoside transporter family (MAL) genes, one inositol transporter family (ITR) gene, two sugar transport-related MFS family genes, and two hypothetical genes. Twelve of the 19 genes were differentially expressed after TM-1 treatment. The HXT gene MRS44_012976 exhibited a time-dependent reversal, being significantly downregulated at 18 h but significantly upregulated at 36 h, with an increase of more than 170-fold. Several MAL family genes also showed differential expression patterns after TM-1 treatment (Supplementary Table S7). Other sugar transport-related classes also responded to TM-1 treatment. The ITR gene MRS44_004943 was suppressed in the 18 h treatment group, although its overall expression was low. Among the MFS family genes, MRS44_016813 had the highest expression, maintained high expression across all samples, and was upregulated at 18 h. In contrast, MRS44_008620 was strongly suppressed by TM-1 and was significantly downregulated at both 18 and 36 h. The two putative hexose transporter homologs showed treatment responses, where MRS44_010803 was barely expressed in the control group but was significantly upregulated after 18 h of treatment, whereas MRS44_006729 was slightly increased at 18 h and downregulated at 36 h (Figures 5A,B). To determine whether these transcriptional changes were functionally associated with fungal growth, five representative genes were selected for knockout from 12 differentially expressed sugar transport-related genes. The selected knockout targets were MRS44_012976, MRS44_016813, MRS44_008620, MRS44_010803, and MRS44_006729, and mutants for these five genes were constructed (Supplementary Figure S7). Phenotypic analysis demonstrated that all five mutants had significantly lower mycelial growth rates than the wild type. Among them, the deletion of MRS44_010803 produced the strongest inhibition of *F. solani* mycelial growth, with a growth inhibition rate of 66.47% (Figure 5C), whereas the deletion of MRS44_016813 produced the weakest effect, with an inhibition rate of 12.56%. Deletion of MRS44_006729, MRS44_008620, and MRS44_012976 reduced mycelial growth, with inhibition rates of 34.64, 48.26, and 39.46%, respectively (Figure 5C). These findings indicate that the selected sugar transport-related genes contribute to normal mycelial growth in *F. solani*. Deletion of these genes affected fungal growth and reduced mycelial development. Together, the transcriptomic and knockout results suggest that TM-1 treatment may affect *F. solani* growth by altering the expression of sugar transport-related genes.

## Discussion

Tobacco root rot is a soil-borne vascular disease that threatens tobacco production because the pathogen persists in soil and plant residues and infects plants through a relatively cryptic process. These features make disease control difficult and increase the need for environmentally sustainable management strategies. Biological control has become an important approach for sustainable plant disease management (Lahlali et al., 2022), and root endophytic microorganisms are increasingly recognized as key components of the plant microbiome. Root endophytes are closely associated with plant health because they can colonize internal plant tissues over extended periods (Carrión et al., 2019; Li et al., 2023). Defining the differences in root endophytic microbiomes between healthy and diseased plants can facilitate the identification of biocontrol resources and disease resistance mechanisms. In the present study, a comparison of healthy and diseased tobacco plants revealed distinct community structures and functional characteristics in root endophytic microbiomes. Guided by these microbiome differences, the antagonistic strain TM-1 was isolated. Transcriptome analysis further indicated that TM-1 markedly altered the transcriptional response of *F. solani* and may restrict mycelial growth by disrupting sugar transporter-related genes. These findings suggest that root endophytic microorganisms are not only associated with the microecological features of tobacco root rot resistance but also provide a useful source for screening functional biocontrol strains. This study provides microbial resources and a mechanistic basis for the biological control of tobacco root rot and supports sustainable disease management strategies based on plant endophytic microbiomes.

The community composition of root endophytic microbiomes differed significantly between healthy and diseased tobacco plants. At the phylum level, Actinobacteria and Bacteroidetes were more abundant in healthy plants. Actinobacteria are well-recognized biocontrol microbial resources because they produce antibiotics, extracellular enzymes, and other antagonistic compounds (Olanrewaju and Babalola, 2019), and they contribute to the suppression of soil-borne pathogens (Lee et al., 2021). In addition, Bacteroidetes have strong capacities for organic matter degradation and complex polysaccharide utilization (McKee et al., 2021), suggesting that their enrichment in healthy plants may reflect greater material transformation activity in the root microenvironment. In contrast, Acidobacteria were significantly enriched in diseased plants. Because Acidobacteria are often associated with oligotrophic or low-pH environments (Kielak et al., 2016; Kalam et al., 2020), their enrichment may indicate an imbalanced diseased root microecosystem. In the present study, the genus-level patterns supported this distinction. PCoA based on Bray–Curtis distances separated healthy and diseased root endophytic bacterial communities along the first principal coordinate, and beneficial bacterial taxa, such as *Chelativorans* and *Egicoccus*, were significantly enriched in healthy plants. These patterns suggest that healthy tobacco roots harbor potentially beneficial bacteria associated with pathogen suppression and plant health, forming a relatively stable disease-suppressive endophytic microecosystem. Such community features may precede disease onset or result from host-mediated recruitment under pathogen infection pressure (Mendes et al., 2011; Van Den Wyngaert et al., 2022). This interpretation is consistent with the “cry for help” hypothesis, which states that plants enhance their defense capacity against pathogen stress by modulating root-associated microbial communities (Rolfe et al., 2019; Liu et al., 2021). However, structural differences alone cannot explain how root endophytic microbiomes contribute to plant disease resistance. Therefore, functional comparisons between healthy and diseased tobacco plants were conducted to identify potential mechanisms associated with disease resistance.

Functional metagenomic analysis extended community-level differences between healthy and diseased plants to the level of microbial function. Nutrient elements, such as nitrogen, phosphorus, and potassium, in the soil and root microenvironment can influence the balance between beneficial microorganisms and pathogens, and may also contribute to disease development (Wang et al., 2020; Cai et al., 2021). In healthy plants, the enrichment of COG categories associated with inorganic ion transport and metabolism (COG_P), amino acid transport and metabolism (COG_E), and carbohydrate transport and metabolism (COG_G) suggested that the root endophytic microbiome may demonstrate stronger nutrient acquisition capacity and metabolic activity. Such functions could provide a competitive advantage in root micro-niches. This interpretation was supported by CAZy analysis, which showed significantly higher relative abundances of multiple carbohydrate-active enzyme-related genes in healthy plants than in diseased plants. The greater potential for carbon source utilization may facilitate colonization by beneficial microorganisms in root tissues, support community stability, and shift the root endophytic microbiome toward a plant health-promoting state. Furthermore, the functional profiles of healthy plants showed several features directly relevant to biological control. KEGG pathway enrichment indicated that the healthy root endophytic microbiome was enriched in secondary metabolite biosynthesis, including non-ribosomal peptide/polyketide biosynthesis, phenazine biosynthesis, and siderophore biosynthesis, together with bacterial chemotaxis, including flagellar assembly and *mcp* genes, and biofilm formation. Non-ribosomal peptide and polyketide biosynthesis, phenazine biosynthesis, and siderophore biosynthesis are closely linked to antimicrobial compound production and iron competition, two common mechanisms used by biocontrol microorganisms. In parallel, the enrichment of flagellar assembly, methyl-accepting chemotaxis protein-related genes, and biofilm formation pathways suggested a stronger chemotactic colonization capacity and greater community stability in root endophytic microorganisms from healthy plants (Mendes et al., 2011). These functional traits support the possibility that healthy tobacco roots could harbor a disease-suppressive endophytic microecosystem. The enrichment of *acdS* provides an additional functional link between healthy root microbiome, stress alleviation, and disease defense. *acdS* gene encodes 1-aminocyclopropane-1-carboxylate deaminase, which degrades ACC, a precursor of plant ethylene biosynthesis, and promotes plant growth under stress conditions. Because some *Enterobacter* strains promote plant growth and enhance biocontrol efficacy through ACC deaminase production, the higher abundance of *acdS* in healthy plants may represent an important functional feature by which the root endophytic microbiome contributes to plant stress alleviation and disease defense. In contrast, the diseased root endophytic microbiome was enriched in pathogen interaction-related pathways, including plant–pathogen interactions and bacterial secretion system-related pathways. This pattern suggests that diseased roots may contain more functional modules associated with pathogen interaction, virulence delivery, or stress adaptation. Thus, tobacco root rot development cannot be determined by a single pathogen but also involves disease-associated shifts in the broader functional structure of the root endophytic microbiome (Guo et al., 2021). Overall, healthy root endophytic microbiomes were more closely associated with nutrient competition, colonization stability, and antimicrobial activity, whereas diseased root microbiomes were more closely linked to pathogen interactions and virulence-related functions. This functional differentiation may constitute an important microecological basis for influencing the occurrence and progression of tobacco root rot.

*Enterobacter* has frequently been associated with plant biocontrol, and its suppressive functions are highly strain-dependent. Reported mechanisms include the induction of systemic resistance (ISR) through salicylic acid- and jasmonic acid-mediated signaling pathways in some strains (Bharathkumar et al., 2009), and the direct inhibition of *F. oxysporum* mycelial growth and sporulation, together with the plant growth promotion through phosphate solubilization, nitrogen fixation, and other beneficial traits (Ordoñez-Flores et al., 2019). These studies have indicated that *Enterobacter* species can contribute to pathogen suppression through multiple mechanisms and that their biocontrol activity should be evaluated within defined strain–host systems. In the present study, *Enterobacter* sp. TM-1 was isolated from healthy tobacco root tissues and demonstrated the strong direct antagonistic activity against *F. solani* in dual-culture plate assays, with an inhibition rate of 62.29%. Its isolation from healthy roots, along with the enrichment of *Enterobacter*-related taxa in the healthy group, suggests that TM-1 may be a functional member of the healthy tobacco root endophytic microbiome involved in pathogen suppression. However, the antifungal mechanisms underlying TM-1-mediated inhibition require further investigation.

Transcriptome analysis provided further insights into the possible mechanism of TM-1-mediated inhibition of *F. solani*. After 18 h of exposure, TM-1 treatment resulted in the differential expression of approximately 6,900 genes, with the significant enrichment of core metabolic pathways, including ribosome biogenesis, amino acid biosynthesis, and carbon metabolism. These changes suggest that TM-1-derived metabolites disrupt fundamental transcriptional and metabolic processes in pathogens. By 36 h, the number of differentially expressed genes had decreased to approximately 3,000, and the GO enrichment signals were markedly reduced. This time-dependent decline suggests that the transcriptional response of *F. solani* weakened under prolonged TM-1-induced stress, potentially reflecting a shift toward a relatively low-activity physiological state.

Sugar transport may be a key process linking this transcriptional disturbance to reduced fungal growth. During infection and colonization, pathogenic fungi depend on sugars as carbon sources, and sugar transporters mediate external carbon uptake required for mycelial growth, competition for host-derived nutrients, and pathogenicity (Wahl et al., 2010; Yuan et al., 2021). TM-1 treatment markedly altered the expression of sugar transporter-related genes in *F. solani*, with 12 of the 19 genes identified in the transcriptome being differentially expressed after treatment. The hexose transporter gene MRS44_012976 showed a pronounced temporal reversal, being significantly downregulated after 18 h but upregulated by more than 170-fold at 36 h. This indicates a disruption in the sugar transporter-related expression balance. Functional validation supported the importance of this gene set in fungal growth. Knockout mutants of five representative sugar transporter-related genes showed significantly reduced mycelial growth, with inhibition rates ranging from 12.56 to 66.47%. The MRS44_010803 knockout caused the strongest inhibition, reaching 66.47%. These results indicate that sugar transporter-related genes are required for normal mycelial growth in *F. solani*. Together, the transcriptomic and gene knockout results suggest that TM-1 may inhibit *F. solani* by altering sugar transporter-related gene expression, disrupting sugar transport and carbon metabolic processes, and reducing nutrient acquisition required for mycelial development.

## Conclusion

In conclusion, this study demonstrated that healthy tobacco roots harbor a distinct endophytic microbiome with enhanced functional potential associated with microbial interaction, colonization, and secondary metabolite biosynthesis. By integrating metagenomic analysis and functional strain isolation, we identified *Enterobacter* sp. TM-1 as a potential biocontrol strain against *F. solani*. Further transcriptomic and functional analyses revealed that TM-1 suppresses pathogen growth by disrupting sugar transport-related processes, highlighting the importance of nutrient acquisition pathways in fungal development. Together, these findings provide new insights into the contribution of root endophytic microbiomes to soil-borne disease suppression and offer valuable microbial resources for sustainable management of tobacco root rot.

## Data Availability

The data presented in the study are deposited in the NCBI Sequence Read Archive (SRA) repository under BioProject accession number PRJNA1512747.
