# Correction: Metagenomic profiling of tobacco root endophytes reveals a disease-suppressive *Enterobacter* strain against *Fusarium solani*

**DOI:** 10.3389/fmicb.2026.1981765

**Published:** 2026-09-16

**Authors:** Frontiers Production Office

**Affiliations:** Frontiers Media SA, Lausanne, Switzerland

**Keywords:** *Enterobacter*, *Fusarium solani*, metagenomics, root endophytes, sugar transporter, tobacco root rot

A correction has been made to the **Affiliations**.

In the original article, Tianmiao Li, Xiaoyu Zhou, and Fei Xiong were incorrectly linked with affiliation 2 Dali Tobacco Company of Yunnan, Dali, China. This should have been Yunnan Academy of Tobacco Agricultural Sciences, Kunming, China. Yunnan Academy of Tobacco Agricultural Sciences, Kunming, China has thus been assigned to affiliation 1, State Key Laboratory of Elemento-Organic Chemistry and Department of Chemical Biology, National Pesticide Engineering Research Center (Tianjin), Nankai University, Tianjin, China to affiliation 2, and Dali Tobacco Company of Yunnan, Dali, China to affiliation 3.

The affiliations for the other authors were correct in the original article, but their numbering has been updated to reflect this change.

The final author list and affiliations thus read as follows:

“Tianmiao Li^1, 2†^, Xiaoyu Zhou^1, 2†^, Fei Xiong^1, 2†^, Tianfu Zhao^3^, Ning Jiang^1^, Yongzhan Cai^4^, Yanxia Hu^3^, Canhua Lu^1^, Yuanhu Xuan^2^^*^ and Xiaotong Gai^1^^*^

^1^Yunnan Academy of Tobacco Agricultural Sciences, Kunming, China

^2^State Key Laboratory of Elemento-Organic Chemistry and Department of Chemical Biology, National Pesticide Engineering Research Center (Tianjin), Nankai University, Tianjin, China

^3^Dali Tobacco Company of Yunnan, Dali, China

^4^Qujing Tobacco Company of Yunnan, Qujing, China

^*^ **Correspondence:**

Yuanhu Xuan

9920230120@nankai.edu.cn

Xiaotong Gai

gaixiaotong0617@163.com

^**†**^These authors have contributed equally to this work and share first authorship”

The numbering has been updated for the other affiliations but the remaining authors still have the same affiliations as in the original publication.

The original **Conflict of Interest** statement was a true reflection of the actual affiliations as given above and has been updated.

The original version of this article has been updated.

