# Supplementary material for: Metagenomic profiling of tobacco root endophytes reveals a disease-suppressive Enterobacter strain against Fusarium solani
Source: Front Microbiol. 2026 Aug 25;17:1924993. doi: 10.3389/fmicb.2026.1924993 (PMC13550168; doi:10.3389/fmicb.2026.1924993)
Supplement: Supplementary file 1 [file Supplementary_file_1.docx]

Figure S1. Beta-diversity analysis of root endophytic bacterial communities in healthy and diseased tobacco plants. (A) Principal coordinate analysis (PCoA) based on Bray–Curtis dissimilarity. Differences between groups were assessed using PERMANOVA (R^2^ = 0.469, P = 0.001). (B) Analysis of similarities (ANOSIM) based on Bray–Curtis dissimilarities, showing significant differences in genus-level community composition between healthy and diseased plants (R = 0.604, P = 0.001).

Figure S2. LEfSe analysis of differentially abundant root endophytic bacterial taxa between healthy and diseased tobacco plants. (A) Cladogram showing the phylogenetic distribution of differentially abundant bacterial taxa. Red nodes indicate taxa enriched in diseased plants, and green nodes indicate taxa enriched in healthy plants. (B) LDA score plot of differentially abundant bacterial taxa between healthy and diseased plants (LDA score > 2, *P* < 0.05).

Figure S3 Number of genes annotated in the CAZy database.

Figure S4 Number of genes annotated in the KEGG database.

Figure S5 Screening of endophytic bacterial isolates with antagonistic activity against *F. solani*. Strain TM-1 showed the strongest inhibitory effect, with an inhibition rate of 62.29%.

Figure S6. Differential gene expression patterns in *F. solani* following exposure to TM-1 filtrate. Volcano plots showing differentially expressed genes after treatment with (A) 10% TM-1 filtrate for 18 h, (B) 20% TM-1 filtrate for 18 h, (C) 10% TM-1 filtrate for 36 h, and (D) 20% TM-1 filtrate for 36 h.

Figure S7. Construction and PCR verification of *F. solani* gene deletion mutants. The schematic diagram illustrates the gene knockout strategy used in *F. solani*. Small arrows indicate the positions of primers used for mutant verification. *HYG* represents the hygromycin B phosphotransferase gene. Scale bar = 500 bp. For each mutant, three primer pairs were used for verification: target-specific primers (PCR-F/R) and primers flanking the 5′ and 3′ homologous recombination regions. Target-specific amplicons were detected only in WT, whereas recombination-specific amplicons were detected only in the corresponding deletion mutants. (A-B) *ΔMRS44_010803* (010803-5-F/R, 010803-3-F/R, and PCR-F/R). (C-D) *ΔMRS44_006729* (006729-5-F/R, 006729-3-F/R, and PCR-F/R). (E-F) *ΔMRS44_008620* (008620-5-F/R, 008620-3-F/R, and PCR-F/R). (G-H) *ΔMRS44_016813* (016813-5-F/R, 016813-3-F/R, and PCR-F/R). (I-J) *ΔMRS44_012976* (012976-5-F/R, 012976-3-F/R, and PCR-F/R).
